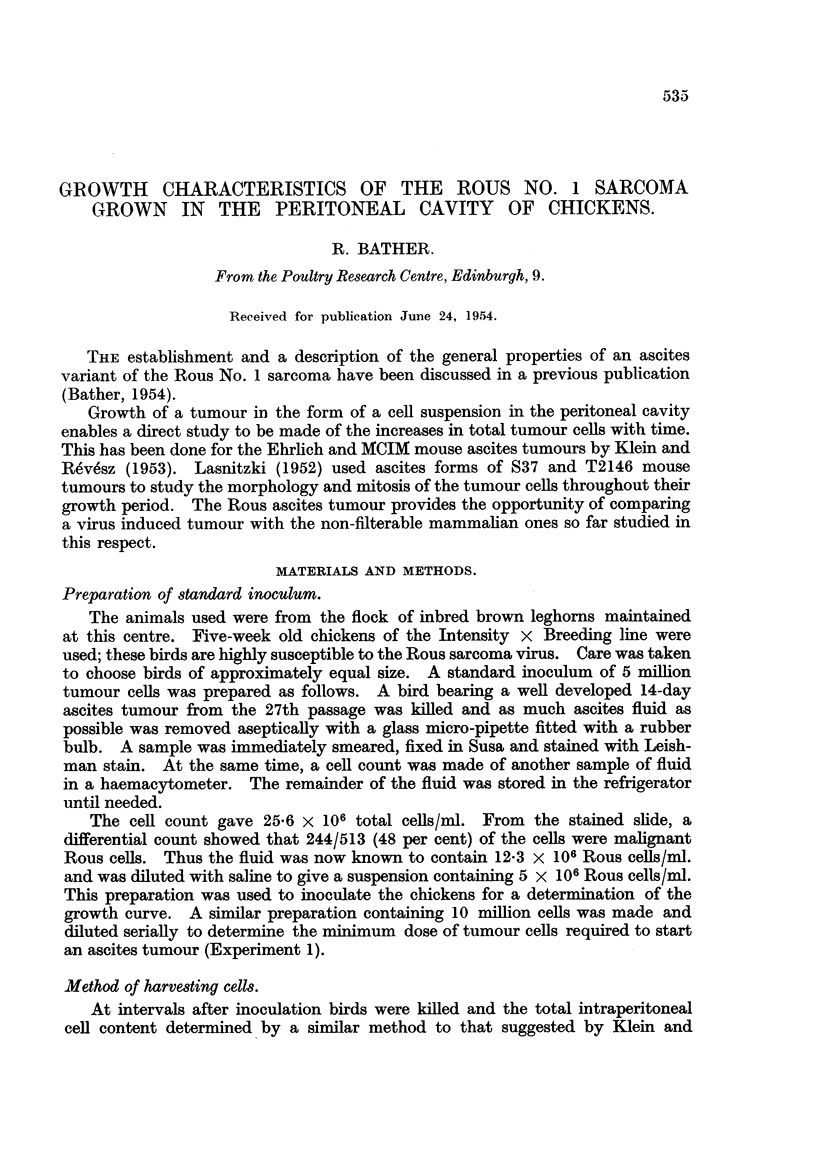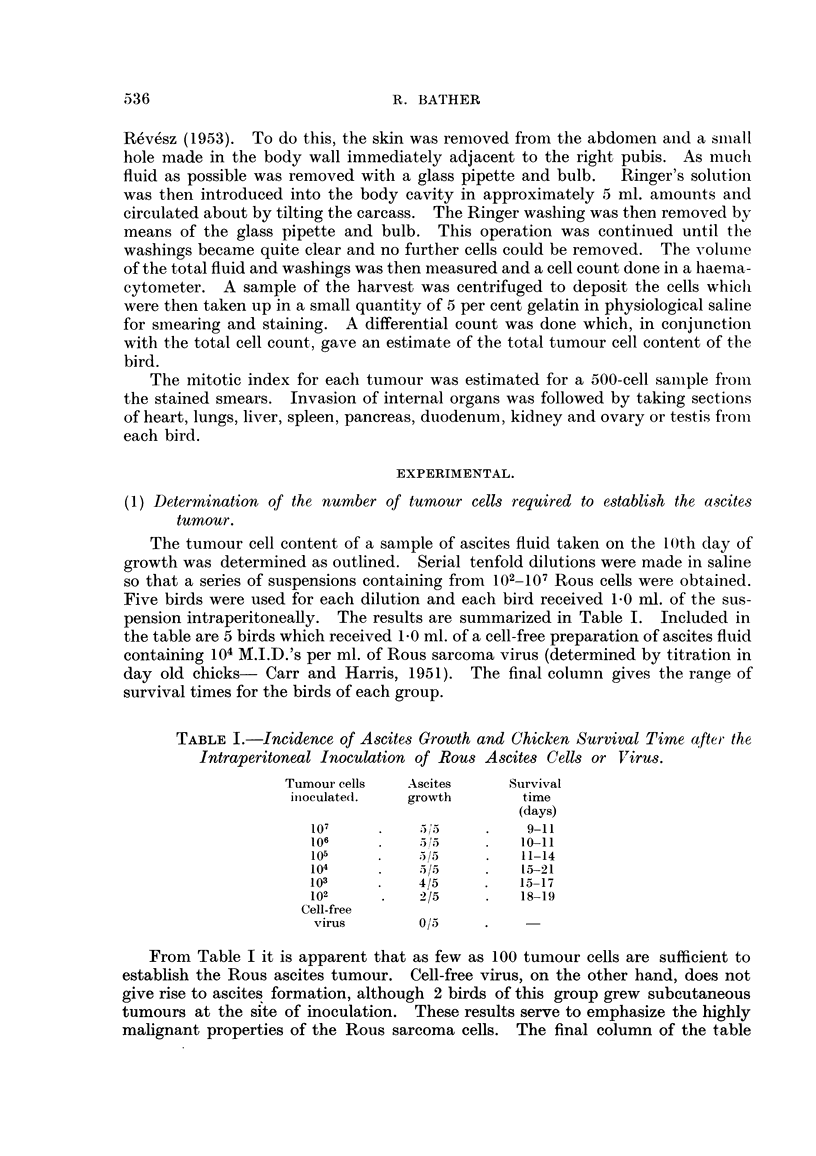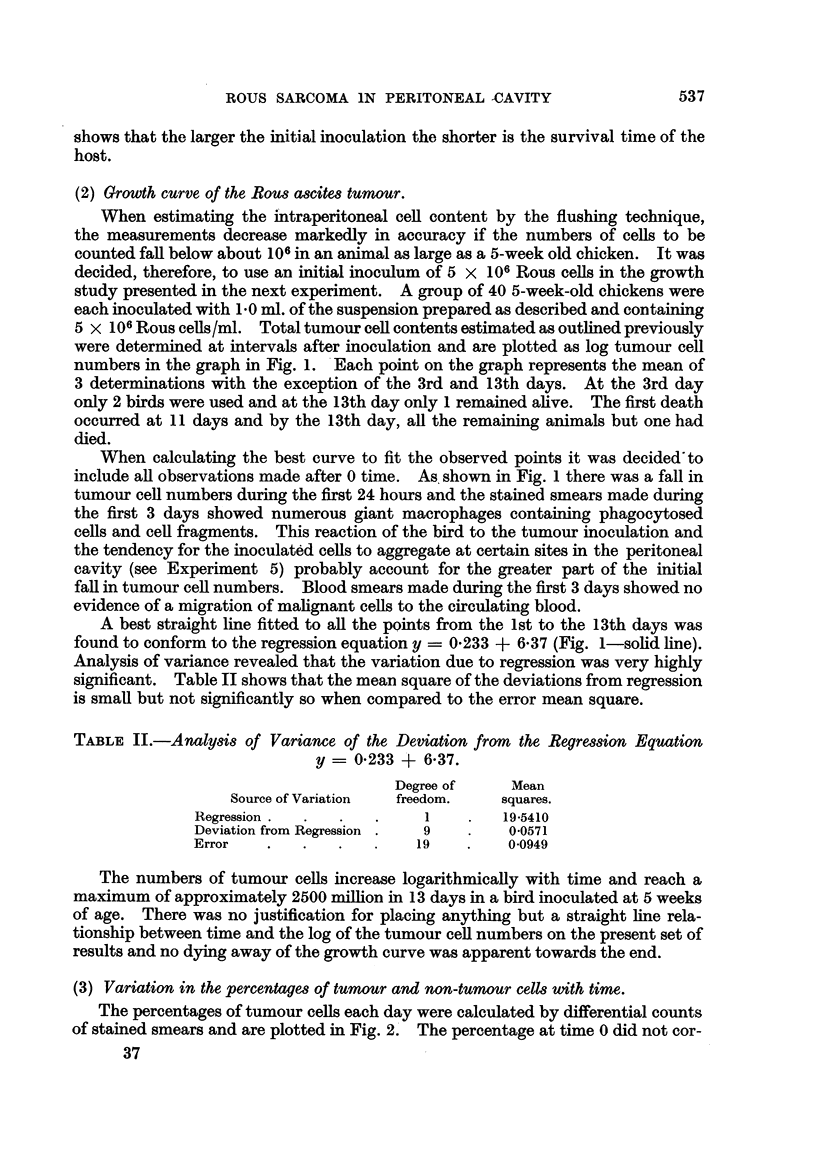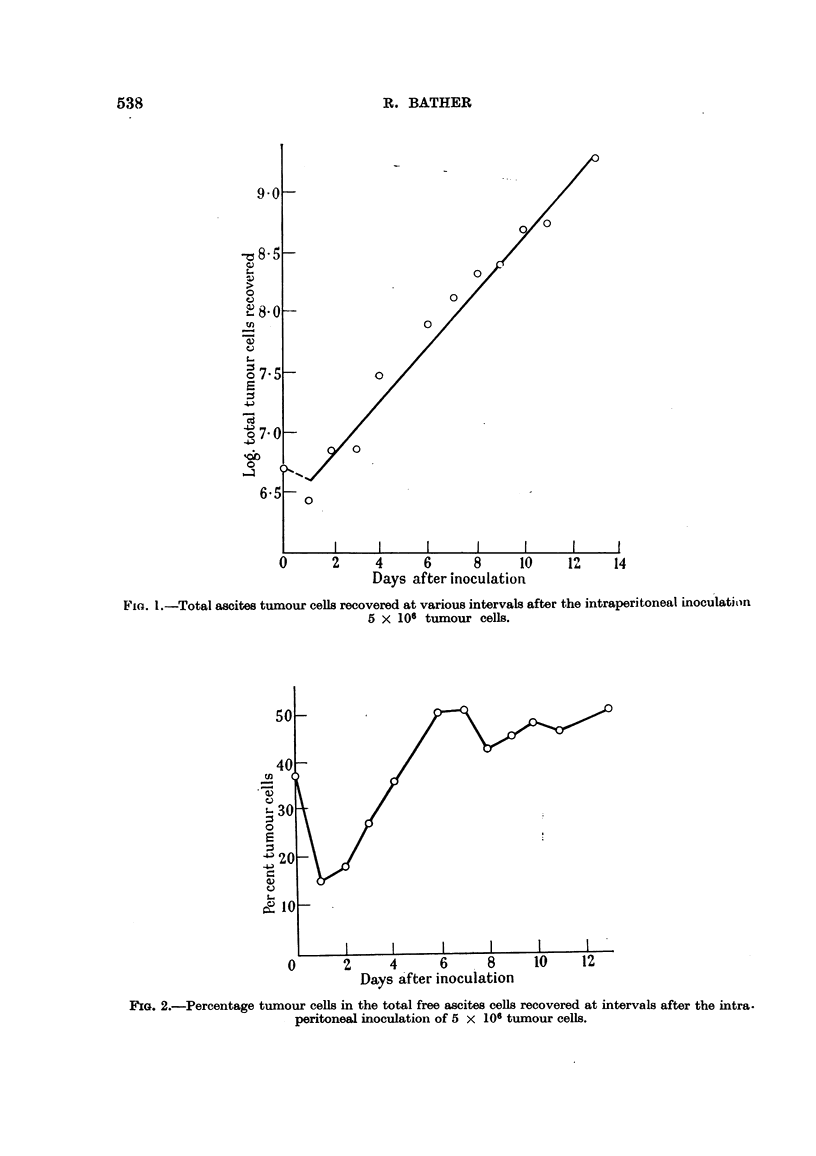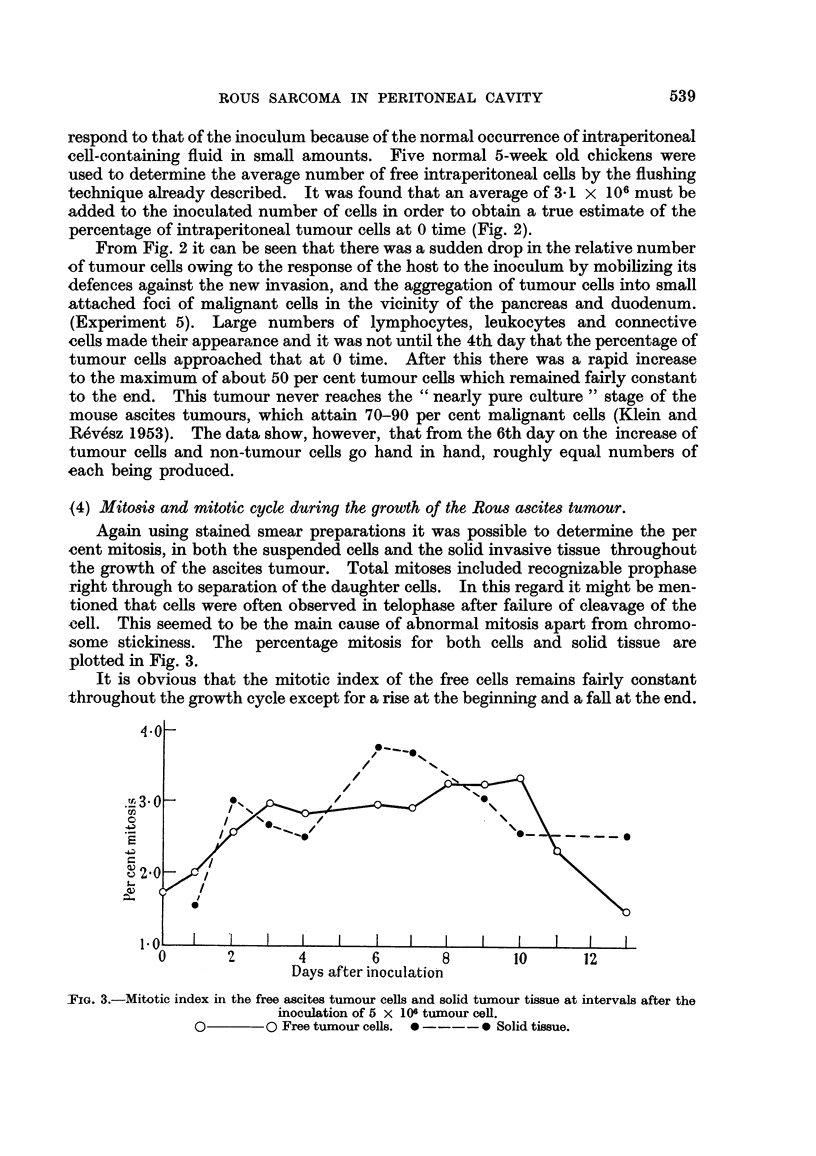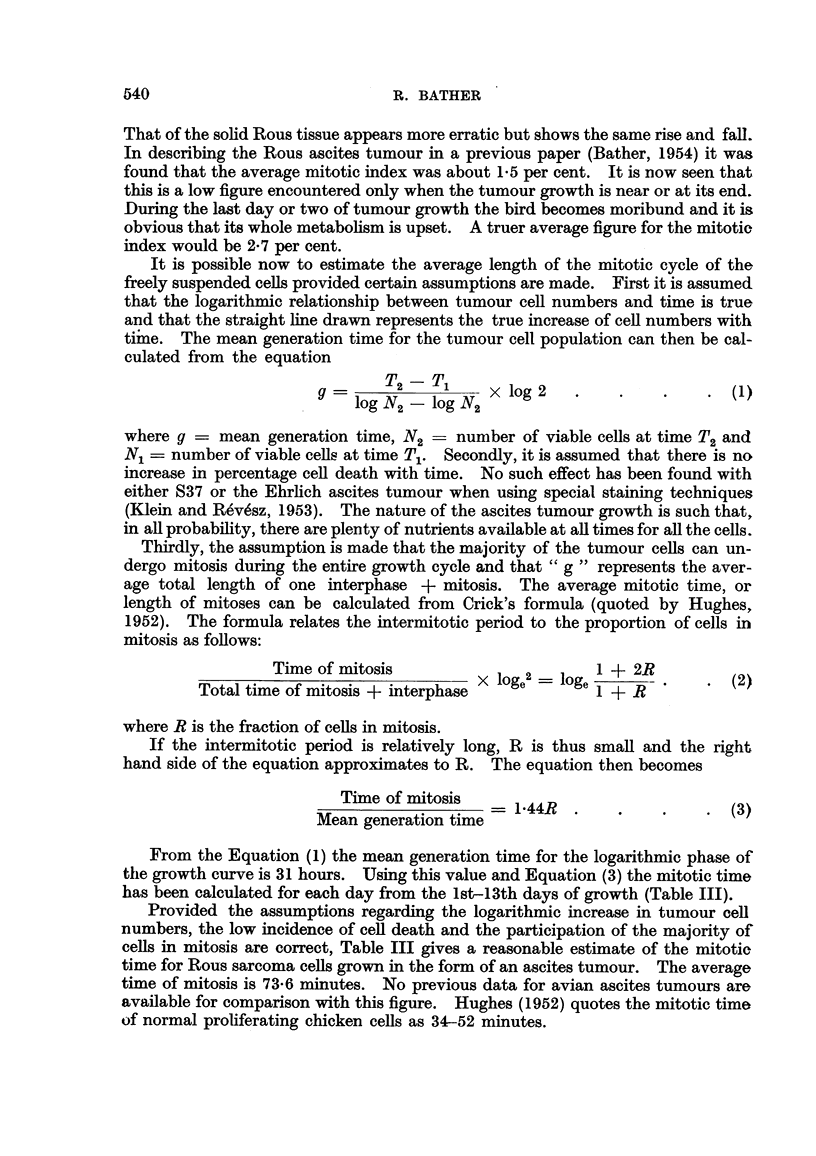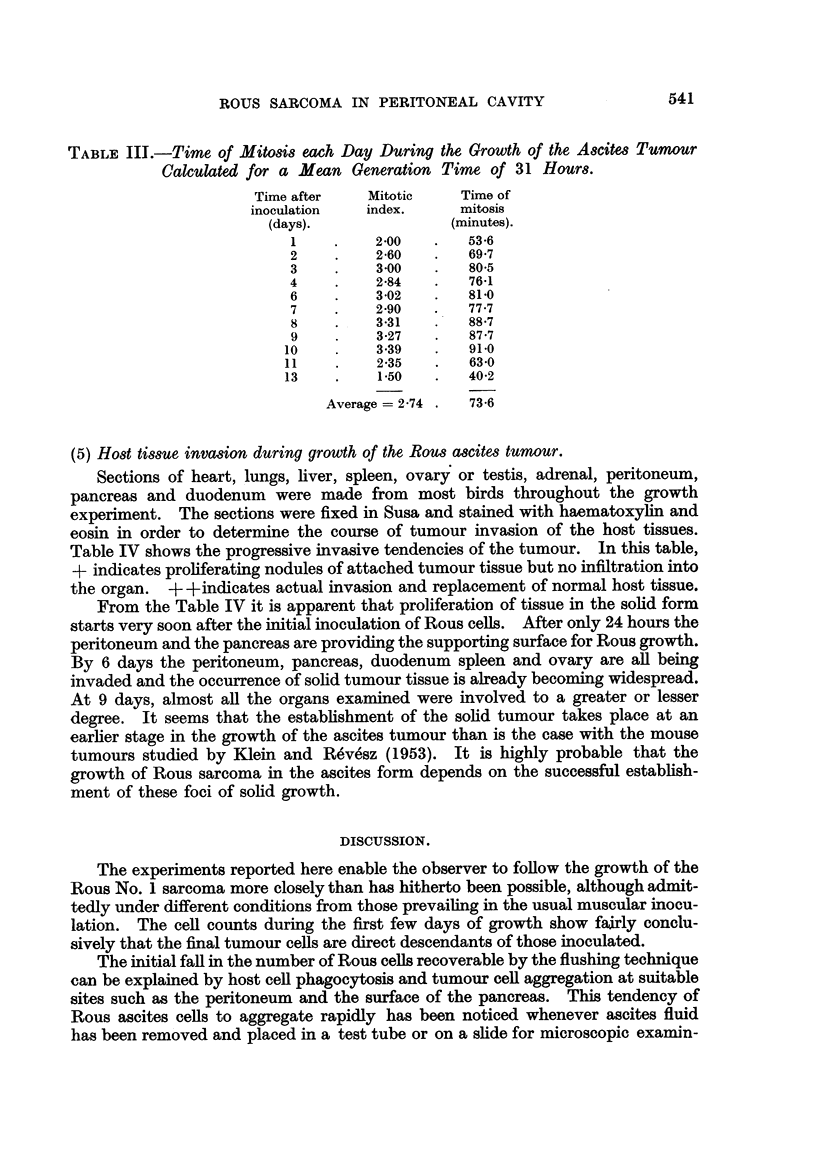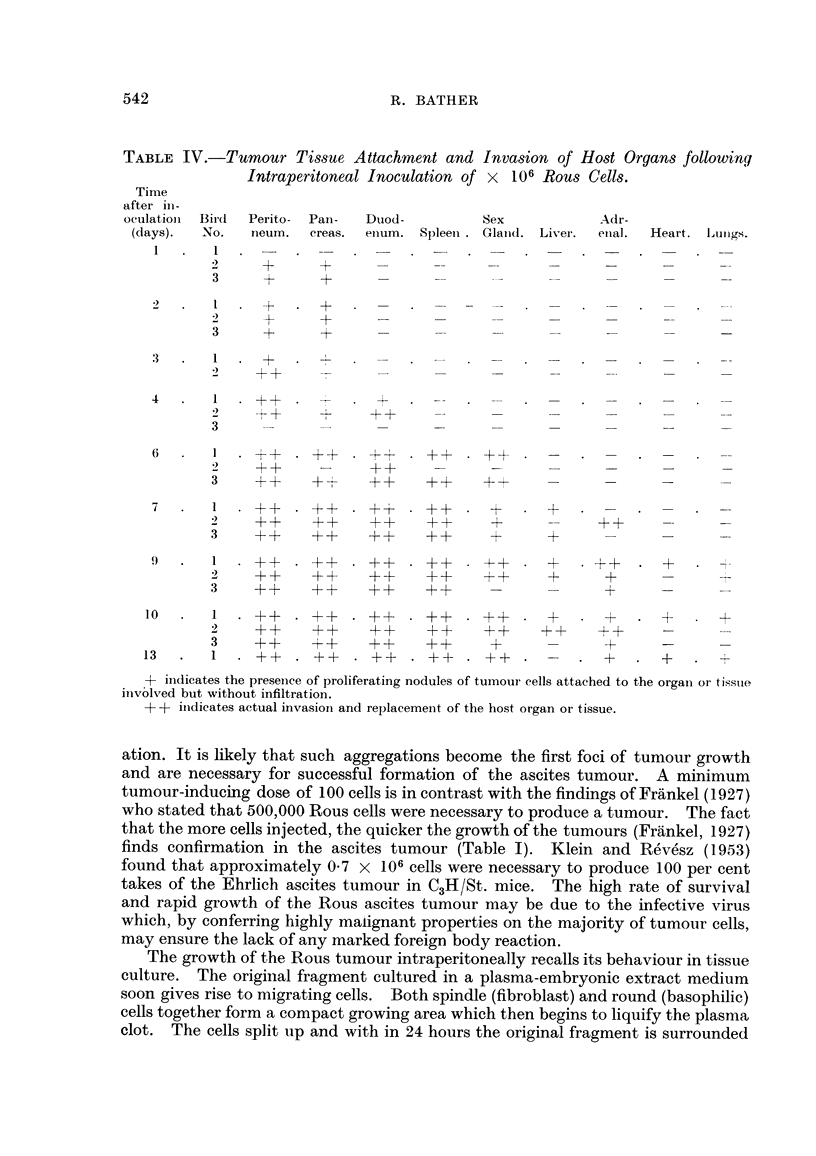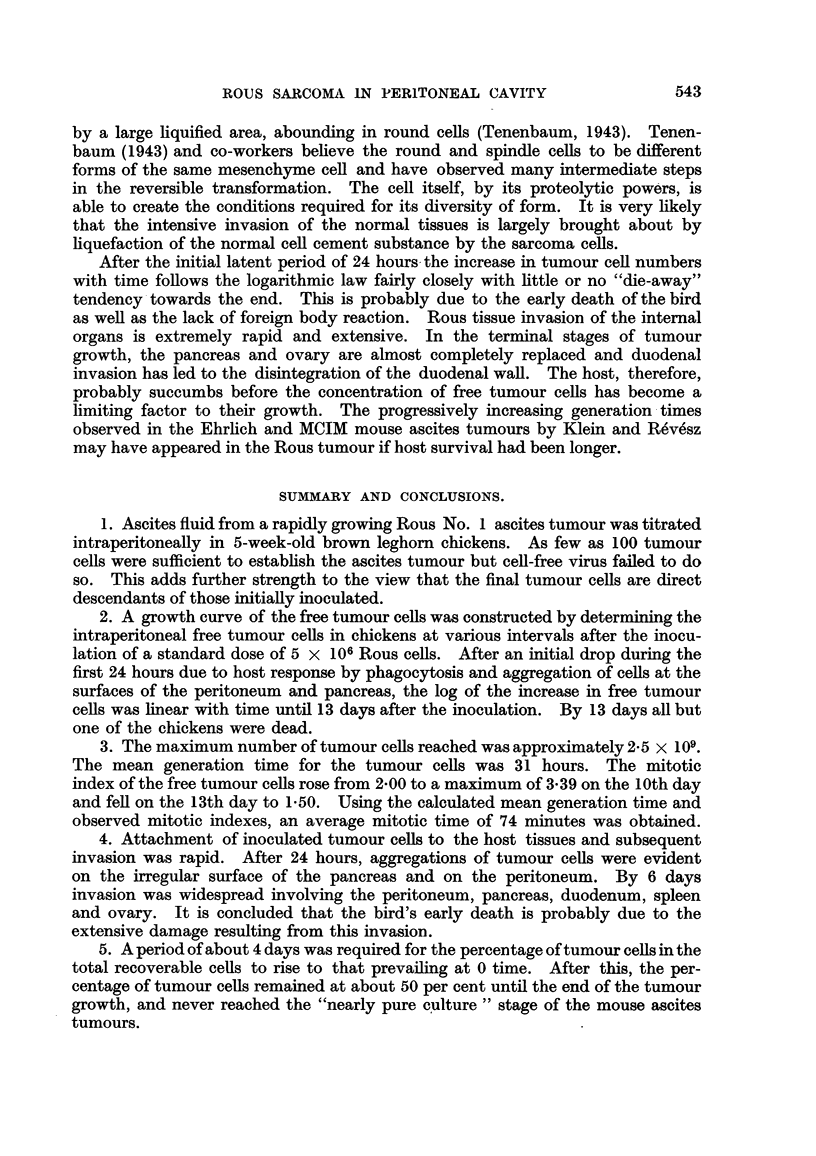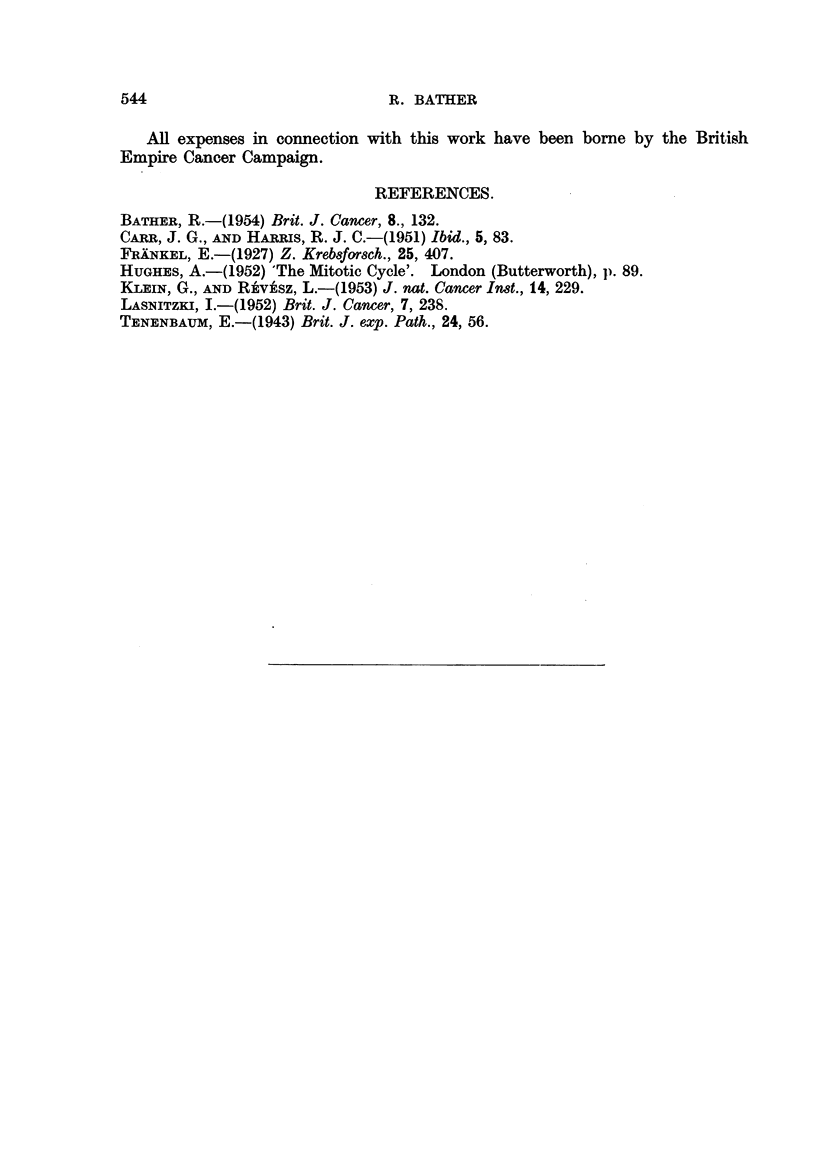# Growth Characteristics of the Rous No. 1 Sarcoma Grown in the Peritoneal Cavity of Chickens

**DOI:** 10.1038/bjc.1954.57

**Published:** 1954-09

**Authors:** R. Bather


### GROWTH CHARACTERISTICS OF THE ROUS NO. 1 SARCOMA GROWN IN THE PERITONEAL CAVITY OF CHICKENS.

R. BATHER.

*From the Poultry Research Centre, Edinburgh, 9.*

Received for publication June 24, 1954.

THE establishment and a description of the general properties of an ascites variant of the Rous No. 1 sarcoma have been discussed in a previous publication (Bather, 1954).

Growth of a tumour in the form of a cell suspension in the peritoneal cavity enables a direct study to be made of the increases in total tumour cells with time. This has been done for the Ehrlich and MCIM mouse ascites tumours by Klein and Révész (1953). Lasnitzki (1952) used ascites forms of S37 and T2146 mouse tumours to study the morphology and mitosis of the tumour cells throughout their growth period. The Rous ascites tumour provides the opportunity of comparing a virus induced tumour with the non-filterable mammalian ones so far studied in this respect.

### MATERIALS AND METHODS.

### *Preparation of standard inoculum.*

The animals used were from the flock of inbred brown leghorns maintained at this centre. Five-week old chickens of the Intensity $\times$ Breeding line were used; these birds are highly susceptible to the Rous sarcoma virus. Care was taken to choose birds of approximately equal size. A standard inoculum of 5 million tumour cells was prepared as follows. A bird bearing a well developed 14-day ascites tumour from the 27th passage was killed and as much ascites fluid as possible was removed aseptically with a glass micro-pipette fitted with a rubber bulb. A sample was immediately smeared, fixed in Susa and stained with Leishman stain. At the same time, a cell count was made of another sample of fluid in a haemacytometer. The remainder of the fluid was stored in the refrigerator until needed.

The cell count gave $25{\cdot}6 \times 10^6$ total cells/ml. From the stained slide, a differential count showed that 244/513 (48 per cent) of the cells were malignant Rous cells. Thus the fluid was now known to contain $12{\cdot}3 \times 10^6$ Rous cells/ml. and was diluted with saline to give a suspension containing $5 \times 10^6$ Rous cells/ml. This preparation was used to inoculate the chickens for a determination of the growth curve. A similar preparation containing 10 million cells was made and diluted serially to determine the minimum dose of tumour cells required to start an ascites tumour (Experiment 1).

### *Method of harvesting cells.*

At intervals after inoculation birds were killed and the total intraperitoneal cell content determined by a similar method to that suggested by Klein and

Révész (1953). To do this, the skin was removed from the abdomen and a small hole made in the body wall immediately adjacent to the right pubis. As much fluid as possible was removed with a glass pipette and bulb. Ringer's solution was then introduced into the body cavity in approximately 5 ml. amounts and circulated about by tilting the carcass. The Ringer washing was then removed by means of the glass pipette and bulb. This operation was continued until the washings became quite clear and no further cells could be removed. The volume of the total fluid and washings was then measured and a cell count done in a haemacytometer. A sample of the harvest was centrifuged to deposit the cells which were then taken up in a small quantity of 5 per cent gelatin in physiological saline for smearing and staining. A differential count was done which, in conjunction with the total cell count, gave an estimate of the total tumour cell content of the bird.

The mitotic index for each tumour was estimated for a 500-cell sample from the stained smears. Invasion of internal organs was followed by taking sections of heart, lungs, liver, spleen, pancreas, duodenum, kidney and ovary or testis from each bird.

### EXPERIMENTAL.

(1) *Determination of the number of tumour cells required to establish the ascites tumour.*

The tumour cell content of a sample of ascites fluid taken on the 10th day of growth was determined as outlined. Serial tenfold dilutions were made in saline so that a series of suspensions containing from $10^2$–$10^7$ Rous cells were obtained. Five birds were used for each dilution and each bird received 1·0 ml. of the suspension intraperitoneally. The results are summarized in Table I. Included in the table are 5 birds which received 1·0 ml. of a cell-free preparation of ascites fluid containing $10^4$ M.I.D.'s per ml. of Rous sarcoma virus (determined by titration in day old chicks— Carr and Harris, 1951). The final column gives the range of survival times for the birds of each group.

TABLE I.—*Incidence of Ascites Growth and Chicken Survival Time after the Intraperitoneal Inoculation of Rous Ascites Cells or Virus.*

| Tumour cells inoculated. | Ascites growth | Survival time (days) |
|---|---|---|
| $10^7$ | 5/5 | 9–11 |
| $10^6$ | 5/5 | 10–11 |
| $10^5$ | 5/5 | 11–14 |
| $10^4$ | 5/5 | 15–21 |
| $10^3$ | 4/5 | 15–17 |
| $10^2$ | 2/5 | 18–19 |
| Cell-free virus | 0/5 | — |

From Table I it is apparent that as few as 100 tumour cells are sufficient to establish the Rous ascites tumour. Cell-free virus, on the other hand, does not give rise to ascites formation, although 2 birds of this group grew subcutaneous tumours at the site of inoculation. These results serve to emphasize the highly malignant properties of the Rous sarcoma cells. The final column of the table shows that the larger the initial inoculation the shorter is the survival time of the host.

(2) *Growth curve of the Rous ascites tumour.*

When estimating the intraperitoneal cell content by the flushing technique, the measurements decrease markedly in accuracy if the numbers of cells to be counted fall below about $10^6$ in an animal as large as a 5-week old chicken. It was decided, therefore, to use an initial inoculum of $5 \times 10^6$ Rous cells in the growth study presented in the next experiment. A group of 40 5-week-old chickens were each inoculated with 1·0 ml. of the suspension prepared as described and containing $5 \times 10^6$ Rous cells/ml. Total tumour cell contents estimated as outlined previously were determined at intervals after inoculation and are plotted as log tumour cell numbers in the graph in Fig. 1. Each point on the graph represents the mean of 3 determinations with the exception of the 3rd and 13th days. At the 3rd day only 2 birds were used and at the 13th day only 1 remained alive. The first death occurred at 11 days and by the 13th day, all the remaining animals but one had died.

When calculating the best curve to fit the observed points it was decided to include all observations made after 0 time. As shown in Fig. 1 there was a fall in tumour cell numbers during the first 24 hours and the stained smears made during the first 3 days showed numerous giant macrophages containing phagocytosed cells and cell fragments. This reaction of the bird to the tumour inoculation and the tendency for the inoculated cells to aggregate at certain sites in the peritoneal cavity (see Experiment 5) probably account for the greater part of the initial fall in tumour cell numbers. Blood smears made during the first 3 days showed no evidence of a migration of malignant cells to the circulating blood.

A best straight line fitted to all the points from the 1st to the 13th days was found to conform to the regression equation $y = 0{\cdot}233 + 6{\cdot}37$ (Fig. 1—solid line). Analysis of variance revealed that the variation due to regression was very highly significant. Table II shows that the mean square of the deviations from regression is small but not significantly so when compared to the error mean square.

TABLE II.—*Analysis of Variance of the Deviation from the Regression Equation* $y = 0{\cdot}233 + 6{\cdot}37$.

| Source of Variation | Degree of freedom. | Mean squares. |
|---|---|---|
| Regression . . . . | 1 . | 19·5410 |
| Deviation from Regression . | 9 . | 0·0571 |
| Error . . . . . | 19 . | 0·0949 |

The numbers of tumour cells increase logarithmically with time and reach a maximum of approximately 2500 million in 13 days in a bird inoculated at 5 weeks of age. There was no justification for placing anything but a straight line relationship between time and the log of the tumour cell numbers on the present set of results and no dying away of the growth curve was apparent towards the end.

(3) *Variation in the percentages of tumour and non-tumour cells with time.*

The percentages of tumour cells each day were calculated by differential counts of stained smears and are plotted in Fig. 2. The percentage at time 0 did not cor-

FIG. 1.—Total ascites tumour cells recovered at various intervals after the intraperitoneal inoculation 5 × 10[6] tumour cells.

FIG. 2.—Percentage tumour cells in the total free ascites cells recovered at intervals after the intraperitoneal inoculation of 5 × 10[6] tumour cells.

respond to that of the inoculum because of the normal occurrence of intraperitoneal cell-containing fluid in small amounts. Five normal 5-week old chickens were used to determine the average number of free intraperitoneal cells by the flushing technique already described. It was found that an average of $3 \cdot 1 \times 10^6$ must be added to the inoculated number of cells in order to obtain a true estimate of the percentage of intraperitoneal tumour cells at 0 time (Fig. 2).

From Fig. 2 it can be seen that there was a sudden drop in the relative number of tumour cells owing to the response of the host to the inoculum by mobilizing its defences against the new invasion, and the aggregation of tumour cells into small attached foci of malignant cells in the vicinity of the pancreas and duodenum. (Experiment 5). Large numbers of lymphocytes, leukocytes and connective cells made their appearance and it was not until the 4th day that the percentage of tumour cells approached that at 0 time. After this there was a rapid increase to the maximum of about 50 per cent tumour cells which remained fairly constant to the end. This tumour never reaches the " nearly pure culture " stage of the mouse ascites tumours, which attain 70–90 per cent malignant cells (Klein and Révész 1953). The data show, however, that from the 6th day on the increase of tumour cells and non-tumour cells go hand in hand, roughly equal numbers of each being produced.

(4) *Mitosis and mitotic cycle during the growth of the Rous ascites tumour.*

Again using stained smear preparations it was possible to determine the per cent mitosis, in both the suspended cells and the solid invasive tissue throughout the growth of the ascites tumour. Total mitoses included recognizable prophase right through to separation of the daughter cells. In this regard it might be mentioned that cells were often observed in telophase after failure of cleavage of the cell. This seemed to be the main cause of abnormal mitosis apart from chromosome stickiness. The percentage mitosis for both cells and solid tissue are plotted in Fig. 3.

It is obvious that the mitotic index of the free cells remains fairly constant throughout the growth cycle except for a rise at the beginning and a fall at the end.

FIG. 3.—Mitotic index in the free ascites tumour cells and solid tumour tissue at intervals after the inoculation of $5 \times 10^6$ tumour cell.
○———○ Free tumour cells. ● — — — ● Solid tissue.

That of the solid Rous tissue appears more erratic but shows the same rise and fall. In describing the Rous ascites tumour in a previous paper (Bather, 1954) it was found that the average mitotic index was about 1·5 per cent. It is now seen that this is a low figure encountered only when the tumour growth is near or at its end. During the last day or two of tumour growth the bird becomes moribund and it is obvious that its whole metabolism is upset. A truer average figure for the mitotic index would be 2·7 per cent.

It is possible now to estimate the average length of the mitotic cycle of the freely suspended cells provided certain assumptions are made. First it is assumed that the logarithmic relationship between tumour cell numbers and time is true and that the straight line drawn represents the true increase of cell numbers with time. The mean generation time for the tumour cell population can then be calculated from the equation

$$g = \frac{T_2 - T_1}{\log N_2 - \log N_2} \times \log 2 \quad . \quad . \quad . \quad . \quad (1)$$

where $g$ = mean generation time, $N_2$ = number of viable cells at time $T_2$ and $N_1$ = number of viable cells at time $T_1$. Secondly, it is assumed that there is no increase in percentage cell death with time. No such effect has been found with either S37 or the Ehrlich ascites tumour when using special staining techniques (Klein and Révész, 1953). The nature of the ascites tumour growth is such that, in all probability, there are plenty of nutrients available at all times for all the cells.

Thirdly, the assumption is made that the majority of the tumour cells can undergo mitosis during the entire growth cycle and that " g " represents the average total length of one interphase + mitosis. The average mitotic time, or length of mitoses can be calculated from Crick's formula (quoted by Hughes, 1952). The formula relates the intermitotic period to the proportion of cells in mitosis as follows:

$$\frac{\text{Time of mitosis}}{\text{Total time of mitosis + interphase}} \times \log_e{}^2 = \log_e \frac{1 + 2R}{1 + R} \quad . \quad . \quad (2)$$

where $R$ is the fraction of cells in mitosis.

If the intermitotic period is relatively long, R is thus small and the right hand side of the equation approximates to R. The equation then becomes

$$\frac{\text{Time of mitosis}}{\text{Mean generation time}} = 1{\cdot}44R \quad . \quad . \quad . \quad . \quad (3)$$

From the Equation (1) the mean generation time for the logarithmic phase of the growth curve is 31 hours. Using this value and Equation (3) the mitotic time has been calculated for each day from the 1st–13th days of growth (Table III).

Provided the assumptions regarding the logarithmic increase in tumour cell numbers, the low incidence of cell death and the participation of the majority of cells in mitosis are correct, Table III gives a reasonable estimate of the mitotic time for Rous sarcoma cells grown in the form of an ascites tumour. The average time of mitosis is 73·6 minutes. No previous data for avian ascites tumours are available for comparison with this figure. Hughes (1952) quotes the mitotic time of normal proliferating chicken cells as 34–52 minutes.

TABLE III.—*Time of Mitosis each Day During the Growth of the Ascites Tumour Calculated for a Mean Generation Time of* 31 *Hours.*

| Time after inoculation (days). | Mitotic index. | Time of mitosis (minutes). |
|---|---|---|
| 1 | 2·00 | 53·6 |
| 2 | 2·60 | 69·7 |
| 3 | 3·00 | 80·5 |
| 4 | 2·84 | 76·1 |
| 6 | 3·02 | 81·0 |
| 7 | 2·90 | 77·7 |
| 8 | 3·31 | 88·7 |
| 9 | 3·27 | 87·7 |
| 10 | 3·39 | 91·0 |
| 11 | 2·35 | 63·0 |
| 13 | 1·50 | 40·2 |
| | Average = 2·74 | 73·6 |

(5) *Host tissue invasion during growth of the Rous ascites tumour.*

Sections of heart, lungs, liver, spleen, ovary or testis, adrenal, peritoneum, pancreas and duodenum were made from most birds throughout the growth experiment. The sections were fixed in Susa and stained with haematoxylin and eosin in order to determine the course of tumour invasion of the host tissues. Table IV shows the progressive invasive tendencies of the tumour. In this table, + indicates proliferating nodules of attached tumour tissue but no infiltration into the organ. ++indicates actual invasion and replacement of normal host tissue.

From the Table IV it is apparent that proliferation of tissue in the solid form starts very soon after the initial inoculation of Rous cells. After only 24 hours the peritoneum and the pancreas are providing the supporting surface for Rous growth. By 6 days the peritoneum, pancreas, duodenum spleen and ovary are all being invaded and the occurrence of solid tumour tissue is already becoming widespread. At 9 days, almost all the organs examined were involved to a greater or lesser degree. It seems that the establishment of the solid tumour takes place at an earlier stage in the growth of the ascites tumour than is the case with the mouse tumours studied by Klein and Révész (1953). It is highly probable that the growth of Rous sarcoma in the ascites form depends on the successful establishment of these foci of solid growth.

### DISCUSSION.

The experiments reported here enable the observer to follow the growth of the Rous No. 1 sarcoma more closely than has hitherto been possible, although admittedly under different conditions from those prevailing in the usual muscular inoculation. The cell counts during the first few days of growth show fairly conclusively that the final tumour cells are direct descendants of those inoculated.

The initial fall in the number of Rous cells recoverable by the flushing technique can be explained by host cell phagocytosis and tumour cell aggregation at suitable sites such as the peritoneum and the surface of the pancreas. This tendency of Rous ascites cells to aggregate rapidly has been noticed whenever ascites fluid has been removed and placed in a test tube or on a slide for microscopic examin-

TABLE IV.—*Tumour Tissue Attachment and Invasion of Host Organs following Intraperitoneal Inoculation of* × $10^6$ *Rous Cells.*

| Time after inoculation (days). | Bird No. | Peritoneum. | Pancreas. | Duodenum. | Spleen. | Sex Gland. | Liver. | Adrenal. | Heart. | Lungs. |
|---|---|---|---|---|---|---|---|---|---|---|
| 1 | 1 | — | — | — | — | — | — | — | — | — |
| | 2 | + | + | — | — | — | — | — | — | — |
| | 3 | + | + | — | — | — | — | — | — | — |
| 2 | 1 | + | + | — | — | — | — | — | — | — |
| | 2 | + | + | — | — | — | — | — | — | — |
| | 3 | + | + | — | — | — | — | — | — | — |
| 3 | 1 | + | + | — | — | — | — | — | — | — |
| | 2 | ++ | + | — | — | — | — | — | — | — |
| 4 | 1 | ++ | + | + | — | — | — | — | — | — |
| | 2 | ++ | + | ++ | — | — | — | — | — | — |
| | 3 | — | — | — | — | — | — | — | — | — |
| 6 | 1 | ++ | ++ | ++ | ++ | ++ | — | — | — | — |
| | 2 | ++ | — | ++ | — | — | — | — | — | — |
| | 3 | ++ | ++ | ++ | ++ | ++ | — | — | — | — |
| 7 | 1 | ++ | ++ | ++ | ++ | + | + | — | — | — |
| | 2 | ++ | ++ | ++ | ++ | + | — | ++ | — | — |
| | 3 | ++ | ++ | ++ | ++ | + | + | — | — | — |
| 9 | 1 | ++ | ++ | ++ | ++ | ++ | + | ++ | + | + |
| | 2 | ++ | ++ | ++ | ++ | ++ | + | + | — | + |
| | 3 | ++ | ++ | ++ | ++ | — | — | + | — | — |
| 10 | 1 | ++ | ++ | ++ | ++ | ++ | + | + | + | + |
| | 2 | ++ | ++ | ++ | ++ | ++ | ++ | ++ | — | — |
| | 3 | ++ | ++ | ++ | ++ | + | — | + | — | — |
| 13 | 1 | ++ | ++ | ++ | ++ | ++ | — | + | + | + |

\+ indicates the presence of proliferating nodules of tumour cells attached to the organ or tissue involved but without infiltration.

++ indicates actual invasion and replacement of the host organ or tissue.

ation. It is likely that such aggregations become the first foci of tumour growth and are necessary for successful formation of the ascites tumour. A minimum tumour-inducing dose of 100 cells is in contrast with the findings of Fränkel (1927) who stated that 500,000 Rous cells were necessary to produce a tumour. The fact that the more cells injected, the quicker the growth of the tumours (Fränkel, 1927) finds confirmation in the ascites tumour (Table I). Klein and Révész (1953) found that approximately $0{\cdot}7 \times 10^6$ cells were necessary to produce 100 per cent takes of the Ehrlich ascites tumour in $C_3H$/St. mice. The high rate of survival and rapid growth of the Rous ascites tumour may be due to the infective virus which, by conferring highly malignant properties on the majority of tumour cells, may ensure the lack of any marked foreign body reaction.

The growth of the Rous tumour intraperitoneally recalls its behaviour in tissue culture. The original fragment cultured in a plasma-embryonic extract medium soon gives rise to migrating cells. Both spindle (fibroblast) and round (basophilic) cells together form a compact growing area which then begins to liquify the plasma clot. The cells split up and with in 24 hours the original fragment is surrounded by a large liquified area, abounding in round cells (Tenenbaum, 1943). Tenenbaum (1943) and co-workers believe the round and spindle cells to be different forms of the same mesenchyme cell and have observed many intermediate steps in the reversible transformation. The cell itself, by its proteolytic powers, is able to create the conditions required for its diversity of form. It is very likely that the intensive invasion of the normal tissues is largely brought about by liquefaction of the normal cell cement substance by the sarcoma cells.

After the initial latent period of 24 hours the increase in tumour cell numbers with time follows the logarithmic law fairly closely with little or no "die-away" tendency towards the end. This is probably due to the early death of the bird as well as the lack of foreign body reaction. Rous tissue invasion of the internal organs is extremely rapid and extensive. In the terminal stages of tumour growth, the pancreas and ovary are almost completely replaced and duodenal invasion has led to the disintegration of the duodenal wall. The host, therefore, probably succumbs before the concentration of free tumour cells has become a limiting factor to their growth. The progressively increasing generation times observed in the Ehrlich and MCIM mouse ascites tumours by Klein and Révész may have appeared in the Rous tumour if host survival had been longer.

### SUMMARY AND CONCLUSIONS.

1. Ascites fluid from a rapidly growing Rous No. 1 ascites tumour was titrated intraperitoneally in 5-week-old brown leghorn chickens. As few as 100 tumour cells were sufficient to establish the ascites tumour but cell-free virus failed to do so. This adds further strength to the view that the final tumour cells are direct descendants of those initially inoculated.

2. A growth curve of the free tumour cells was constructed by determining the intraperitoneal free tumour cells in chickens at various intervals after the inoculation of a standard dose of $5 \times 10^6$ Rous cells. After an initial drop during the first 24 hours due to host response by phagocytosis and aggregation of cells at the surfaces of the peritoneum and pancreas, the log of the increase in free tumour cells was linear with time until 13 days after the inoculation. By 13 days all but one of the chickens were dead.

3. The maximum number of tumour cells reached was approximately $2{\cdot}5 \times 10^9$. The mean generation time for the tumour cells was 31 hours. The mitotic index of the free tumour cells rose from 2·00 to a maximum of 3·39 on the 10th day and fell on the 13th day to 1·50. Using the calculated mean generation time and observed mitotic indexes, an average mitotic time of 74 minutes was obtained.

4. Attachment of inoculated tumour cells to the host tissues and subsequent invasion was rapid. After 24 hours, aggregations of tumour cells were evident on the irregular surface of the pancreas and on the peritoneum. By 6 days invasion was widespread involving the peritoneum, pancreas, duodenum, spleen and ovary. It is concluded that the bird's early death is probably due to the extensive damage resulting from this invasion.

5. A period of about 4 days was required for the percentage of tumour cells in the total recoverable cells to rise to that prevailing at 0 time. After this, the percentage of tumour cells remained at about 50 per cent until the end of the tumour growth, and never reached the "nearly pure culture " stage of the mouse ascites tumours.

All expenses in connection with this work have been borne by the British Empire Cancer Campaign.

### REFERENCES.

BATHER, R.—(1954) *Brit. J. Cancer*, **8**., 132.
CARR, J. G., AND HARRIS, R. J. C.—(1951) *Ibid.*, **5**, 83.
FRÄNKEL, E.—(1927) *Z. Krebsforsch.*, **25**, 407.
HUGHES, A.—(1952) 'The Mitotic Cycle'. London (Butterworth), p. 89.
KLEIN, G., AND RÉVÉSZ, L.—(1953) *J. nat. Cancer Inst.*, **14**, 229.
LASNITZKI, I.—(1952) *Brit. J. Cancer*, **7**, 238.
TENENBAUM, E.—(1943) *Brit. J. exp. Path.*, **24**, 56.